# Potential Environmental Risk Characteristics of PCB Transformation Products in the Environmental Medium

**DOI:** 10.3390/toxics9090213

**Published:** 2021-09-07

**Authors:** Minghao Li, Wei He, Hao Yang, Shimei Sun, Yu Li

**Affiliations:** 1 The Moe Key Laboratory of Resources and Environmental Systems Optimization, North China Electric Power University, Beijing 102206, China; lmh66@ncepu.edu.cn (M.L.); 120202232011@ncepu.edu.cn (W.H.); 120212232080@ncepu.edu.cn (H.Y.); 2 School of Emergency Science and Engineering, Jilin Jianzhu University, Changchun 130119, China

**Keywords:** environmental risk, polychlorinated biphenyls, transformation products, three-dimensional quantitative structure-activity relationships, transformation pathways

## Abstract

The complementary construction of polychlorinated biphenyl (PCB) phytotoxicity and the biotoxicity 3D-QSAR model, combined with the constructed PCB environmental risk characterization model, was carried out to evaluate the persistent organic pollutant (POP) properties (toxicity (phytotoxicity and biotoxicity), bioconcentration, migration, and persistence) of PCBs and their corresponding transformation products (phytodegradation, microbial degradation, biometabolism, and photodegradation). The transformation path with a significant increase in environmental risks was analyzed. Some environmentally friendly PCB derivatives, exhibiting a good modification effect, and their parent molecules were selected as precursor molecules. Their transformation processes were simulated and evaluated for assessing the environmental risks. Some transformation products displayed increased environmental risks. The environmental risks of plant degradation products of the PCBs in the environmental media showed the maximum risk, indicating that the potential risks of the transformation products of the PCBs and their environmentally friendly derivatives could not be neglected. It is essential to further improve the ability of plants to degrade their transformation products. The improvement of some degradation products for environmentally friendly PCB derivatives indicates that the theoretical modification of a single environmental feature cannot completely control the potential environmental risks of molecules. In addition, this method can be used to analyze and evaluate environmentally friendly PCB derivatives to avoid and reduce the potential environmental and human health risks caused by environmentally friendly PCB derivatives.

## 1. Introduction

Polychlorinated biphenyls (PCBs) are considered persistent organic pollutants (POPs) that spread into the environment in large quantities. The global production of PCBs is estimated to be approximately 1 to 2 million tons, out of which 0.2–0.4 million tons have produced environmental hazards [1]. The degradation or metabolism of PCBs in the environment can occur by using a variety of pathways. For instance, PCBs can be degraded to benzoic acid products by using the expression of dioxygenase degradation genes in tobacco and Arabidopsis plants [2,3,4]. Microorganisms can reduce the dechlorination of PCBs and degrade highly chlorinated PCBs to less-chlorinated ones [5]. In addition, microorganisms can also degrade PCBs by using cytochrome P450 enzymes (CYP450) in vivo to produce hydroxy PCB products with hydroxyl groups (OH-PCBs) [6]. The metabolism of PCBs by organisms can produce polychlorinated biphenyl methane sulfonate (MeSO_2_-PCB) through reactions such as oxidative substitution [7]. Under natural light radiation conditions, PCBs in the environment can absorb ultraviolet light and undergo direct photodegradation, and optically active chlorine atoms can break bonds in order to produce dechlorination products [8].

Among the multiple pathways of PCB transformation, the degradation or metabolites such as OH-PCBs and MeSO_2_-PCBs are also persistent and biotoxic [9,10,11,12]. The DNA-damaging effect of PCB-180 on rat liver was due to its metabolite 3′-OH-PCB180, which indicates that the hydroxyl metabolites of PCBs might show a higher potential for toxicity than the parent compound [13]. OH-PCBs exhibit the potential to interfere with estrogen levels in animals and humans and even in infants, which adversely affects the developmental and reproductive functions in animals and humans [14]. In addition, MeSO_2_ PCBs showed toxic effects and displayed stronger environmental persistence than the parent PCBs and easily enriched the food chain [15]. Therefore, further studies on the environmental risk characteristics of PCB degradation or metabolites can provide theoretical references for PCB pollution control.

This paper evaluates the environmental risks of PCB transformation products by using the following four pathways: plant degradation, microbial degradation, biometabolism, and photodegradation. The international evaluation criteria of the POPs primarily examine the following four properties of pollutants: toxicity, bioconcentration, persistence and migration. Therefore, these four characteristics were selected for evaluating the environmental risk characteristics of PCB degradation and metabolites. The POP evaluation was not defined as the toxicity evaluation receptor of pollutants. Considering that the degradation and metabolites of PCBs show estrogen interference toxicity to organisms and toxicity to plants at the same time, the phytotoxicity and biotoxicity (estrogen toxicity) of the PCBs are selected in the scope of the toxicity evaluation. In addition, the potential risk characteristics of the PCB transformation products in environmental media are primarily evaluated by using the 3D-QSAR model of the environmental risk characteristics of the PCBs.

## 2. Materials and Methods

### 2.1. Data Sources

(1) The data sources of the PCB environmental characteristics

The Stockholm Convention determines whether a chemical substance can be classified as a POP by using the following four characteristics: toxicity (phytotoxicity and biotoxicity), bioconcentration, migration, and persistence. Each characteristic requires its own characteristic parameters for evaluating the degree of each characteristic. Bioconcentration factors (BCFs) are used to represent biological enrichment. Bioconcentration as a component of risk assessment determines a meaningful BCF value for hazardous substances. It indicates the potential hazardous capacity of a substance and is the basis for assessing environmental and human risks [16]. As the evaluation criterion for the retention time of PCBs in environmental media, the half-life (*t*_1/2_) was used. The larger the *t*_1/2_ of a PCB is, the longer the retention time in the environmental media will be [17]. The octanol air partition coefficient (*K*_OA_) can respond to the migration ability of PCBs to some extent. As the *K*_OA_ decreases, PCBs easily volatilize into the air [18]. When PCBs enter the plant body, they can cause the oxidation of the cell membrane and several organelles, inhibit peroxidase activity, and damage the health of the plant body [19,20,21]. Therefore, this paper selected the total score of the PCB interactions with peroxidase for characterizing the phytotoxicity of the PCBs and with estrogen receptors for representing the biotoxicity (estrogen toxicity) of the PCBs. The 3D-QSAR models of the PCB bioconcentration [16], migration [17], and persistence [18] refer to the existing models. The 3D-QSAR models of PCB phytotoxicity and biotoxicity were constructed in this paper. The structures of the receptor enzyme for phytotoxicity (1CCK) and estrogen receptor enzyme (3MDJ) for estrogen toxicity were derived from the Protein Data Bank (http://www1.rcsb.org; accessed on 15 February 2021).

(2) The data sources of transformation pathways and transformation products of PCBs in the environmental media

**Figure 1:**
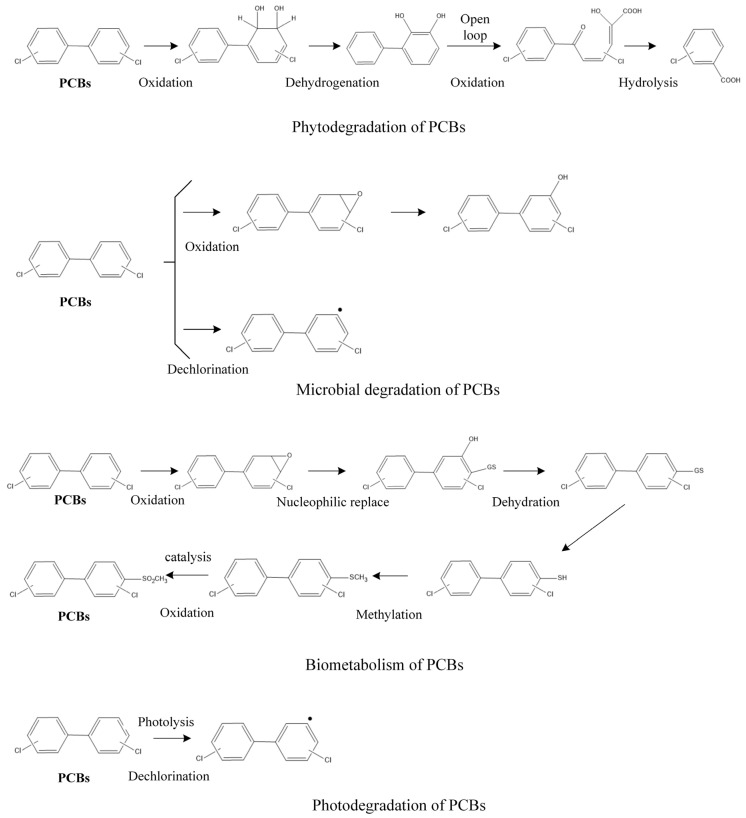
Schematic diagram of the four degradation or metabolic pathways of the PCBs.

The literature review summarized the following four transformation pathways of the PCBs [7,22,23,24,25]: plant degradation pathway, microbial degradation pathway, biometabolism pathway, and photodegradation pathway (Figure 1). The pathway for the degradation of PCBs by plants is usually a preferential attack on the non-chlorine-substituted benzene ring by dioxygenases. However, the oxidation reaction can also occur on the chlorine-substituted ring if there is no barrier at the 2 and 3 carbon positions. For instance, first, PCBs are oxidized by dioxygenases at the 2 and 3 carbon positions in order to produce 2,3-dihydro dihydroxy PCB products; then, 2,3-dihydro dihydroxy PCBs undergo dehydrogenation reactions in order to produce 2,3-dihydroxy PCB products; thereafter, 2,3-dihydroxy PCBs undergo the meta-ring opening reaction by oxidation; finally, the resulting meta-ring opening mixture is hydrolyzed in order to produce polychlorinated benzoic acid [26]. In the microbial degradation pathway, PCBs can be oxidized to epoxides through oxidation, primarily by attacking the meta- and para-substitution sites of PCBs. The epoxidation products after meta- and para-oxidation can be directly metabolized into hydroxy PCBs by adding hydroxyl groups. In addition, PCBs can also undergo a reductive dechlorination reaction, in which the main reduction sites are meta and para, and the ortho-reaction is relatively less [27]. First, the biometabolism of PCBs is catalyzed by enzymes to produce epoxidation intermediates and carry out the methylation of PCBs by the nucleophilic reaction, dehydration, and methylation. Finally, PCBs with methyl benzenesulfonic acid were synthesized by catalytic oxidation [7]. The essence of the photodegradation of PCBs is the change of molecular energy under the action of light radiation from a low-energy state to a high-energy state, chemical bond breaking, and chemical reaction. PCBs can absorb ultraviolet light directly [28]. Fifty PCB degradation or metabolism products are summarized in Table 1.

### 2.2. 3D-QSAR Model Construction of PCB Toxicity (Phytotoxicity and Estrogen Toxicity)

In this paper, we used SYBYL-X 2.0 software for molecular structure mapping. The PCBs were studied by using the Minimize module of SYBYL-X 2.0 software. The energy convergence was limited to 0.005 kJ/mol by using the Powell conjugate gradient method with the Gasteiger-Huckel charge, and the Tripos force field was selected for 10,000 iterations. The optimized molecules were stored in the database, and the PCBs with the highest environmental risk values in the PCB samples were used as the common skeleton for superposition.

StockholOpen was used as the molecular library of the training set. The environmental risk values of some PCBs were input into the database in turn, and the model parameters were automatically calculated using the calculate properties function. In order to establish the relationship between the structure and biological activity of the target compounds, the partial least-squares (PLS) analysis was used. By using the leave-one-out (L-O-O) method, the training set compounds were cross-validated, and the cross-validation coefficient *q*^2^ and the best principal component *n* were calculated. Then, by using the non-cross-validation function (No Validation), the regression analysis was performed. Finally, to ensure a reliable 3D-QSAR estimation model for the PCB risk characteristics, the non-cross-validation coefficient *r*^2^, standard deviation SEE, and the test value *F* were calculated [16].

## 3. Results

### 3D-QSAR Model Construction and Evaluation of PCB Toxicity (Phytotoxicity and Estrogen Toxicity)

**Figure 2:**
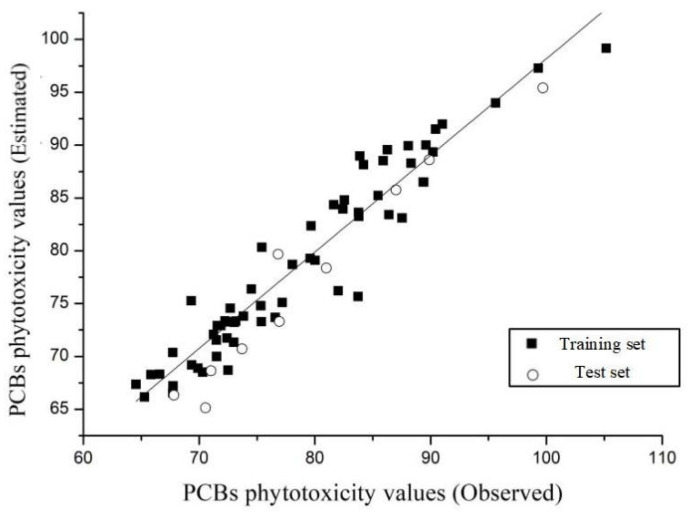
The plot of the observed vs. estimated phytotoxicity values of PCBs by using the 3D-QSAR models.

Based on the CoMFA method using the total score of 70 PCBs docked with the 1CCK enzyme as the dependent variable and their molecular structures as the independent variables, the 3D-QSAR model for the phytotoxicity of the PCBs was constructed. In this process, 60 PCBs were randomly selected as the training set, and the remaining 10 molecules were selected as the test set. Based on the CoMFA method, the results showed that the best principal component *n* and the cross-validation coefficient *q*^2^ of the constructed 3D-QSAR model were 8 and 0.695 (*q*^2^ > 0.5), respectively, which indicates that the model exhibited good estimation ability. The non-cross-validation coefficient *R*^2^, the standard deviation SEE, and the test value *F* were estimated as 0.914 (*R*^2^ > 0.9), 2.854, and 67.952, respectively, which indicates that the constructed model fulfilled the stability requirements and exhibited good fitting and estimation abilities [32]. Based on the CoMFA method, Figure 2 shows the linear fit plots of the experimental and estimated values of the 3D-QSAR model for the phytotoxicity of PCBs. The results showed that all the data were concentrated around the trend line, and the *R*-value was 0.956, which indicates that the linear fit between the experimental and estimated values was good. The model exhibited a high internal estimative power [32]. This model can be used for estimating the phytotoxicity values of PCBs and their derivatives.

Based on the CoMFA method, the constructed 3D-QSAR model of phytotoxicity in the PCBs estimated 70 PCBs with known experimental values. The results showed that the relative error between the experimental and estimated values of the phytotoxicity in 70 PCBs was less than 10% [16]. The estimated values of the phytotoxicity in 209 PCBs are shown in Table 2.

**Figure 3:**
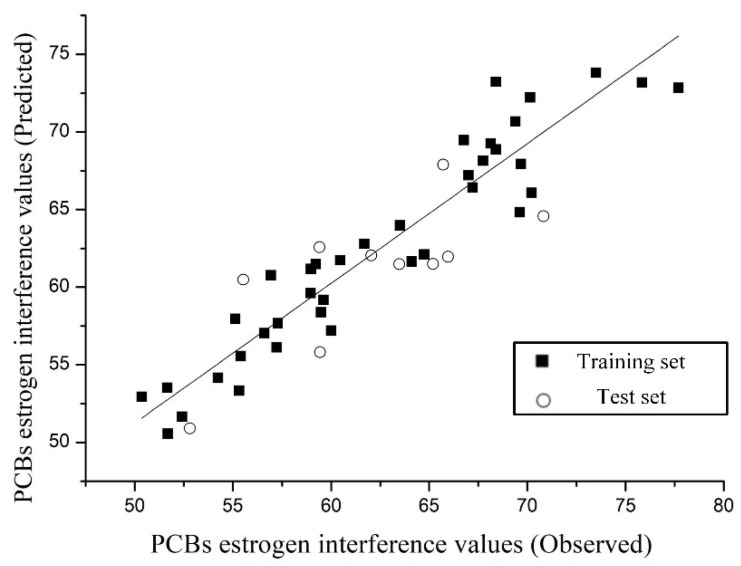
The plot of the observed vs. estimated estrogen interference values by the 3D-QSAR models.

In addition, the total-score of 48 PCBs docked with the 3GZX enzyme were selected for representing the estrogen toxicity, and 38 PCBs and 10 PCBs were randomly selected in the training and test sets of the model for constructing the 3D-QSAR model of estrogen toxicity. Based on the CoMFA method, the results showed that the 3D-QSAR model of estrogen toxicity in PCBs showed a good estimation ability by using the best principal component *n* with a value of 7 and the cross-validation coefficient *q*^2^ with a value of 0.671 (*q*^2^ > 0.5). The constructed model fulfilled the stability requirements and exhibited a good fitting ability (the non-cross-validation coefficient *R*^2^ of 0.90 (*R*^2^ > 0.9), the standard deviation SEE, and test values *F* of 2.509 and 38.578, respectively) [32]. The experimental and estimated values of the test and training sets of the estrogen toxicity model in PCBs were linearly fitted (Figure 3). As shown in Figure 3, all the data were concentrated near the trend line with an *R*-value of 0.949, which indicated a high correlation coefficient and estimate capability for the linear fit of the relationship between the experimental and estimated values [14]. This model can be used for estimating the estrogen toxicity values of PCBs and their derivatives (Figure 3). The 3D-QSAR model of estrogen toxicity in PCBs was used for estimating the estrogen toxicity values of 209 PCBs. The relative errors between the experimental and estimated values of the estrogen toxicity in 48 PCBs were less than 10% [16].

## 4. Discussion

### 4.1. The Estimation of the Environmental Risk Characteristics of PCB Transformation Products in Environmental Media

To determine the degradation path of PCB degradation and transformation products with the greatest environmental risk, a total of 50 PCB transformation products were estimated by using the phytodegradation pathway, microbial degradation pathway, biometabolism pathway, and photodegradation pathway of the PCBs. Five kinds of environmental risk characteristics (phytotoxicity, estrogen toxicity, bioconcentration, persistence, and migration) of the PCB transformation products were evaluated. As shown in Table S1, the ranges of the phytotoxicity, estrogen toxicity, bioconcentration, persistence, and migration for the different PCB transformation products were as follows: for PCB phytodegradation products: −1.36% to 22.92%, −11.01% to 8.32%, 22.08% to 61.26%, 56.87% to 421.71%, and 1.22% to 32.27%, respectively; for PCB microbial aerobic degradation products: −14.46% to 17.93%, −10.48% to 15.48%, −5.40% to 9.50%, −37.71% to 12.31%, and −19.68% to 18.51%, respectively; for PCB microbial anaerobic degradation products: −8.82% to 32.53%, −5.76% to 12.92%, −13.09% to 4.05%, −25.45% to 9.64%, and −19.10% to −3.99%, respectively; for PCB biometabolism products: −8.97% to 19.40%, −8.74% to 1.89%, −4.45% to 19.39%, −5.69% to 42.10%, and −2.92% to 20.42%, respectively; for PCB photodegradation products: 13.35% to 36.06%, 12.63% to 40.28%, −22.93% to −11.85%, −60.70% to −15.36%, and −16.20% to −1.89%, respectively.

**Figure 4:**
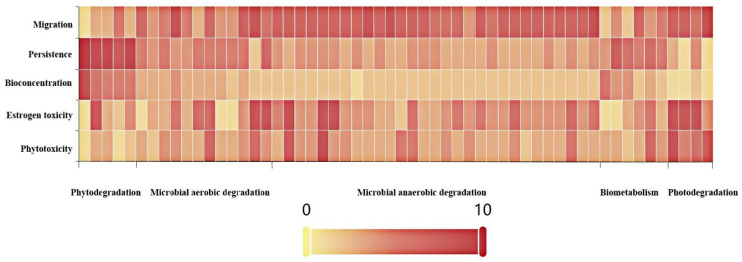
Environmental risk characteristics of the PCB transformation products under different degradation pathways.

Figure 4 is a heat map of the environmental risk characteristics (phytotoxicity, estrogen toxicity, bioconcentration, persistence, and migration) of PCB transformation products under different degradation pathways (the phytodegradation pathway, the microbial degradation pathway, the biometabolism pathway, and the photodegradation pathway). The color of the heat map is divided into 10 levels. The higher the color level, the greater the variation range of environmental risk characteristics of PCBs degradation products. As shown in Figure 4, the region of PCBs plant degradation products has the darkest color. Combined with the data of Table S1, the environmental risk characteristics of the PCB degradation products showed a maximum increase of 421.71%. Therefore, the environmental risk of the PCB plant degradation products was the highest. Improving the degradation of PCBs by plants is of great significance for environmental health. In addition, the change of environmental risk of the microbial products in all the PCB degradation products is relatively small, indicating that microbial degradation methods have little impact on secondary environmental pollution. The microbial anaerobic degradation method has certain advantages over the microbial aerobic degradation method. The phytotoxicity of anaerobic degradation products was higher than that of aerobic degradation products, and other properties were improved than that of the aerobic degradation products.

In summary, the environmental risk characteristics of PCB degradation products were increased in different degrees under different degradation pathways, which, for the future, indicates that the environmental risk characteristics of PCB degradation products should not be neglected.

### 4.2. The Estimation of the Environmental Risk Characteristics of Environmentally Friendly PCB Transformation Products in Plants

**Figure 5:**
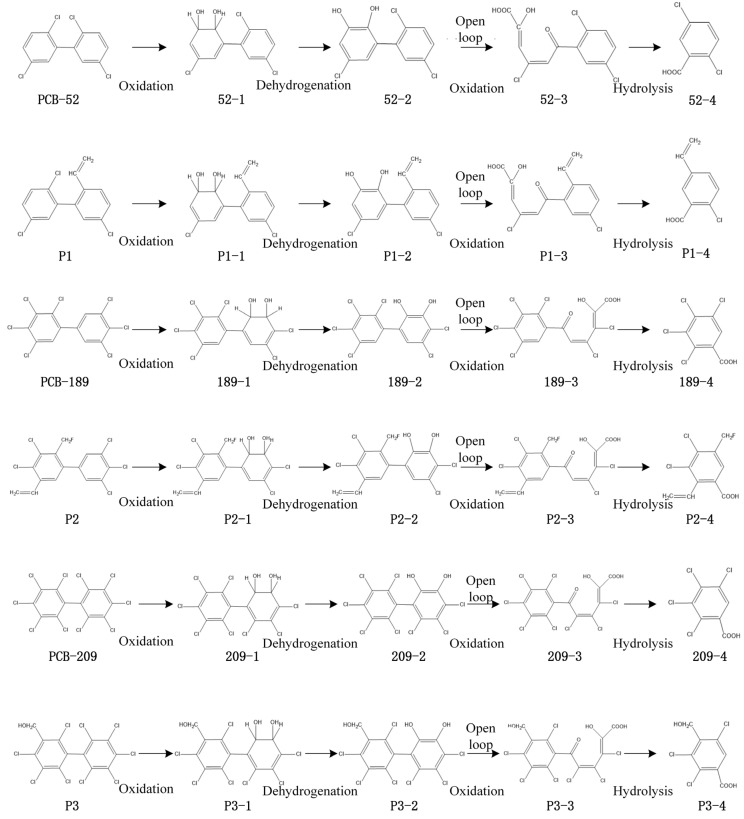
Schematic diagram of the phytodegradation products of three PCBs and their environmentally friendly derivatives.

In this study, the environmental risk of the PCB phytodegradation products was the highest. Environmentally friendly PCB derivatives refer to those PCB molecules whose functions remain unchanged and environmental risk characteristics (such as the representative characteristics of persistent organic pollutants) are improved by the design method of molecular modification. Considering the phytodegradation pathway as an example, some environmentally friendly PCB derivatives were designed in different studies [16,32,33], and their parent molecules (low migration environmentally friendly derivative P1 and the parent molecule PCB-52, low bioconcentration environmentally friendly derivative P2, and the parent molecule PCB-189, and low toxicity environmentally friendly derivative P3 and the parent molecule PCB-209) were selected for analyzing the environmental risks. The specific path inference of the molecules is shown in Figure 5.

The higher the parameter value of migration is, the lower the risk of physical environment will be. The other four environmental risk characteristic parameters showed contrary characteristics. As shown in Table 3, the ranges of phytotoxicity, estrogen toxicity, bioconcentration, persistence, and mobility of the phytodegradation products of the three PCBs (PCB-52, PCB-189, and PCB-209) were −63.71% to 34.98%, −13.84 to 29.36%, −74.97% to 16.03%, −184.02% to 11.43%, and −61.55 to 18.42%, respectively. Though the environmental risk of most of the PCB degradation products has been reduced, the environmental risk of some products is still increasing. The target organism of phytotoxicity, estrogen toxicity, and bioconcentration of the PCBs is focused on the human body. The increased phytotoxicity represents that the transformation products of PCBs, for example, they may enter the human body through the food chain and, finally, increase the threat to human health [34]. The increase of estrogen toxicity also represents that the transformation products of PCBs interfere with the health of the human endocrine system and affect the normal expression of estrogen [35]. The bioconcentration also represents the enrichment ability of the transformation products of PCBs in the human body. The greater the enrichment degree, the stronger the harm to the human body [36]. The persistence effect of the transformation products of PCBs is also implied in the human body and organism in the environment. The long-term existence of the transformation products of PCBs will damage the health of the human body or organism exposed to the environment of PCB metabolites [37], and the migration effect of the transformation products of PCBs also includes the human body or organism far away from the contaminated environment, which represents the long-distance mobility of the transformation products of PCBs in the atmosphere. The research shows that, in Arctic seabirds and Greenland sharks, PCBs were detected at certain concentrations [38,39], indicating that, once the transformation products of PCB conversion products flow into the environment, they will have long-distance migration and cause risks to the environment health. Therefore, as compared to the parent compounds of PCBs, the migration characteristics of phytodegradation products of the three PCBs showed the highest increase in environmental risks, and the variation range was up to 61.55%, which indicates that, when the migration ability of PCB degradation products is improved once they flow into the environment, there is a risk of long-distance migration. The potential risk of long-range migration of the transformation products of PCBs cannot be completely overcome by controlling their parent’s migration capacities.

The variations of phytotoxicity, estrogenic toxicity, bioconcentration, persistence, and migration of the three environmentally friendly PCB derivatives (P1, P2, and P3) of the phytodegradation products were estimated to range from −18.33% to 21.81%, −21.60 to 40.81%, −23.06% to 60.65%, −49.06% to 13.65%, and −22.46 to 117.32%, respectively. Similarly, the environmental risk of some degradation products was observed to increase. As compared to the parent molecules of the environmentally friendly PCB derivatives, their phytodegradation products showed the best bioconcentration performance. The results showed that the risk of bioconcentration of the PCB degradation products was increased. The bioconcentrations of PCBs in breast milk in urban areas in China were 2.66–3.90 pg/g [36] and, in the adipose tissue of Belgians, were 490-ng/g lipid weight [40]. The accumulation of PCBs in the human body increases with age and, hence, can indirectly cause visceral [41], endocrine [42], and reproductive diseases [43]. Therefore, the control of the bioconcentration ability of environmentally friendly PCB derivatives should not be neglected.

**Figure 6:**
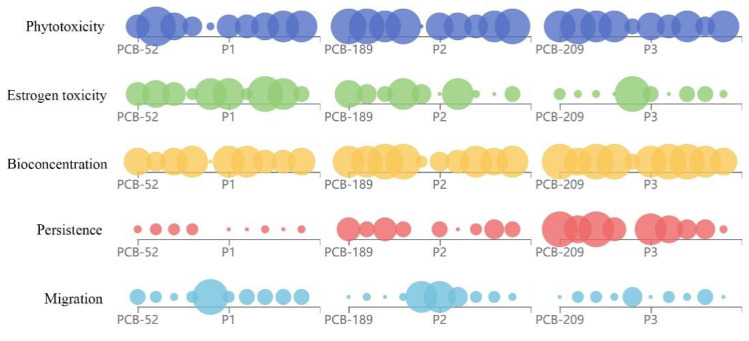
Changes in the environmental characteristics of PCB conversion products.

Figure 6 is an effect diagram that represents the changes in the environmental characteristics of PCB conversion products. In Figure 6, the size of the sphere represents the activity value of each molecule. Comparing the size of the sphere, the final phytodegradation product of P1 represents the low mobility derivative of PCB-52 (Figure 6). As compared to the final phytodegradation product of PCB-52, the migration of the final P1 phytodegradation product is still lower. This result is found to be consistent with the design concept [16,32,33]. In addition, the estrogenic toxicity and the migration of the final P1 phytodegradation product also showed a significant improvement as compared to the final PCB-52 phytodegradation product. The improvement in estrogen toxicity and migration were estimated as 19.52% and 156%, respectively. As compared to the final degradation product of low bioconcentration derivative P2, the final PCB-189 degradation products showed no improvement in terms of the bioconcentration properties and improvement in the estrogen toxicity and migration properties. It was observed that the phytotoxicity of P3 was improved compared to PCB-209, but the environmental risk of the final product was increased. The environmental risk of P3 was higher as compared to PCB-209 in biotoxicity, but the environmental risk of the final product was significantly improved by 26.14%. In addition, the migration of the final P3 product was improved up to 37.68%.

In summary, the environmental risks of the final degradation products of environmentally friendly PCB derivatives P1 and P2 showed improvements, agreeing with the modification results. However, some degradation products still showed an increase in environmental risks, indicating that the environmental risk control of the PCB degradation products and their environmentally friendly derivatives cannot be neglected. The potential environmental risk of PCBs cannot be completely controlled by the theoretical modification of single environmental characteristics. Therefore, the environmental risks of the transformed products of the environmentally friendly PCB derivatives are also required to be considered.

### 4.3. The Validation of the Total Score and Its Estimated Value of PCBs and Their Products Containing Different Chemical Structure

The phytotoxicity value and estrogen toxicity value of parent PCBs are derived from the total score after docking with the corresponding enzymes. Taking the phytotoxicity and estrogen toxicity as examples, we calculated the total score of the PCBs and their metabolites containing different chemical structures (such as −OH, −SO_2_CH_3_, etc.) in the manuscript and analyzed the correlation and relative error between the total score and the estimated value by the 3D-QSAR model (Table 4). The results showed that the correlation coefficient r between the total cost of 33 molecular estrogen toxicities and their predicted values was 0.547, which met the correlation coefficient test standard (i.e., when *p* = 0.001, the correlation limit value r_0_ is 0.539). However, the correlation coefficient r between the total cost of the phytotoxicity of 33 molecules and their estimated values was only 0.369, the correlation was relatively lower, which only met the correlation coefficient test standard when *p* = 0.05 (the correlation limit value r_0_ is 0.339). Most of the relative errors were within the allowable range, only one-third of the molecules having a relative error more than 10%.

The above results indicate that, indeed, the 3D-QSAR model constructed by the parent PCBs data is slightly lesser accurate in estimating PCBs with different chemical structures and their metabolites. Most of the relative errors are negative, indicating that the 3D-QSAR model has indeed underestimated the toxicity of PCBs with different chemical structures and their metabolites, which should actually be higher than those estimated values. However, since the purpose is to measure the environment risk of PCB metabolites on a relative scale, the overall trend of toxicity of the PCB metabolites is consistent with the estimation in this study. The overall analysis of the results is reasonable in the manuscript.

## 5. Conclusions

In this paper, the transformation pathways of PCBs (phytodegradation, microbial degradation, biometabolism, and photodegradation) were derived. The constructed 3D-QSAR models were used for estimating the POP characteristics (toxicity (phytotoxicity and biotoxicity), bioconcentration, migration, and persistence) of PCBs and their transformed products. In addition, for the environmental risk evaluation of PCBs and their environmentally friendly derivative transformation products, the plant degradation pathway with the highest environmental risk increase was selected. The environmental risk of some PCBs and their derivative degradation products was observed to be increased, which indicated that the environmental risk control of PCBs and their environmentally friendly derivative degradation products could not be neglected. The potential environmental risk of PCBs cannot be completely controlled by theoretical modification considering single environmental characteristics. Therefore, the environmental risks of the transformed products of environmentally friendly PCBs are also required to be considered.

## Figures and Tables

**Table 1:** Summary of the degradation or metabolites of the PCBs [7,24,29,30,31].

NO.	Degradation Pathway	Parent Molecule	Degradation or Metabolites
1	Plant degradation	PCB-3	4-CBA
2		PCB-4	2-CBA
3		PCB-5	2,3-CBA
4		PCB-11	3-CBA
5		PCB-31	2,5-CBA
6	Microbial aerobic degradation	PCB-97	4′-OH-CB97
7		PCB-101	4′-OH-CB101
8		PCB-107	4-OH-CB107
9		PCB-109	4-OH-CB109
10		PCB-118	3-OH-CB118
11		PCB-148	4-OH-CB148
12		PCB-153	3-OH-CB153
13		PCB-162	4-OH-CB162
14		PCB-172	4′-OH-CB172
15		PCB-187	4-OH-CB187
16		PCB-199	4′-OH-CB199
17		PCB-202	4-OH-CB202
18	Microbial anaerobic degradation	PCB-90	PCB-49 PCB-68
19		PCB-91	PCB-51
20		PCB-92	PCB-52 PCB-72
21		PCB-95	PCB-53
22		PCB-99	PCB-47
23		PCB-101	PCB-49
24		PCB-102	PCB-51
25		PCB-130	PCB-90
26		PCB-132	PCB-91
27		PCB-135	PCB-94
28		PCB-137	PCB-90 PCB-99
29		PCB-138	PCB-99
30		PCB-146	PCB-90
31		PCB-147	PCB-91
32		PCB-149	PCB-102
33		PCB-151	PCB-95
34		PCB-153	PCB-99
35		PCB-154	PCB-100
36		PCB-170	PCB-130 PCB-137 PCB-138
37		PCB-174	PCB-149
38		PCB-180	PCB-153 PCB-146
39		PCB-183	PCB-154
40		PCB-187	PCB-149
41	Biometabolism	PCB-49	3′-MeSO2-CB49
42		PCB-64	4-MeSO2-CB64
43		PCB-70	3-MeSO2-CB70
44		PCB-110	3-MeSO2-CB110
45		PCB-149	4-MeSO2-CB149
46		PCB-174	4-MeSO2-CB174
47	Photodegradation	PCB-47	PCB-15
48		PCB-40	PCB-11
49		PCB-101	PCB-70
50		PCB-171	PCB-35

**Table 2:** Estimated phytotoxicity and estrogen interference values of the PCBs by using the 3D-QSAR models.

PCBs (IUPAC)	Phytotoxicity			Estrogen Toxicity			PCBs (IUPAC)	Phytotoxicity			Estrogen Toxicity		
	Estd.	Obs.	Relative Error (%)	Estd.	Obs.	Relative Error (%)		Estd.	Obs.	Relative Error (%)	Estd.	Obs.	Relative Error (%)
0	80.694			71.424			105	91.091			65.655		
1	80.325	75.426	−6.50	70.958			106	81.145			69.353		
2	84.363	81.624	−3.36	70.674	69.396	−1.84	107	84.562			70.567		
3	83.949	82.426	−1.85	67.889	65.720 ^a^	−3.30	108	89.361			69.117		
4	73.802	73.822	0.03	63.977	63.512	−0.73	109	67.583			57.749		
5	79.090	80.007	1.15	71.672			110	68.633	71.019 ^a^	3.36	61.736	60.469	−2.10
6	83.632	83.766	0.16	71.588			111	82.886			73.235	68.404	−7.06
7	83.259	83.786	0.63	67.399			112	63.621			62.042	62.049 ^a^	0.01
8	85.749	87.006 ^a^	1.44	68.852			113	70.030			64.786		
9	78.359	80.980 ^a^	3.24	72.131			114	83.185			66.746		
10	71.729	72.398	0.92	64.205			115	58.217			55.372		
11	83.118	87.533	5.04	73.814	73.504	−0.42	116	61.924			58.568		
12	88.281	88.323	0.05	67.752			117	67.104			57.672	57.287	−0.67
13	89.938	88.073	−2.12	67.219	67.002	−0.32	118	88.524	85.897	−3.06	65.011		
14	81.876			72.630			119	67.740			57.761		
15	89.550	86.282	−3.79	65.043			120	86.663			68.555		
16	75.274	69.318	−8.59	64.841			121	71.060			55.813		
17	72.967			60.820			122	90.103			68.870	68.399	−0.69
18	72.089	71.252	−1.17	63.389			123	93.991	95.620	1.70	64.795		
19	69.084			56.327			124	88.268			69.449		
20	82.354	79.668	−3.37	72.228	70.152	−2.96	125	75.464			60.670		
21	82.154			68.549			126	99.156	105.165	5.71	66.748		
22	84.490			69.539			127	92.611			71.565		
23	76.212	82.007	7.07	72.676			128	68.328	66.586	−2.62	58.372	59.488	1.88
24	63.846			64.834	69.621	6.88	129	71.759			62.271		
25	86.493	89.403	3.25	67.989			130	74.548	72.689	−2.56	62.574	59.408 ^a^	−5.33
26	81.368			72.709			131	62.701			52.006		
27	74.870			64.884			132	66.789			54.858		
28	88.612	89.893 ^a^	1.42	65.302			133	73.554			65.404		
29	80.237			67.876			134	62.993			56.028		
30	70.770			60.778	56.932	−6.76	135	64.861			59.012		
31	83.420	86.392	3.44	70.012			136	64.381			50.250		
32	65.574			61.050			137	67.115			58.501		
33	89.357	90.196	0.93	68.142	67.742	−0.59	138	68.889	69.900	1.45	57.865		
34	87.497			71.727			139	68.238			50.422		
35	86.138			69.104			140	60.491			48.887		
36	91.986	91.012	−1.07	73.519			141	74.464			61.583		
37	91.435			64.331			142	70.237			52.663		
38	92.285			69.477	66.772	−4.05	143	66.244			54.364		
39	91.511	90.431	−1.19	69.204			144	64.648			55.168		
40	73.327	73.154	−0.24	65.302			145	74.108			61.477	63.476 ^a^	3.15
41	68.109			61.187			146	75.031			60.764		
42	74.542			61.114			147	65.471			52.282		
43	74.076			64.081			148	70.797			52.261		
44	66.061			65.927			149	68.510			54.156	54.240	0.16
45	60.125			56.469			150	79.539			46.136		
46	66.153	65.282	−1.33	54.948			151	67.629			57.030	56.600	−0.76
47	65.818			57.198	60.010	4.69	152	77.851			63.524		
48	65.849			60.330			153	66.585	67.731	1.69	57.174		
49	71.324			61.956	65.967 ^a^	6.08	154	67.694			50.534		
50	68.467			53.531	51.652	−3.64	155	74.516			64.116		
51	63.434			52.778			156	86.505	89.360	3.19	66.754		
52	67.203	67.751	0.81	64.519			157	93.025			65.739		
53	68.356			66.008			158	69.996			60.483	55.534 ^a^	−8.91
54	66.777			60.121			159	84.798	82.536	−2.74	69.399		
55	85.426			69.076			160	65.245			58.509		
56	88.107			68.780			161	71.927			56.126	57.228	1.93
57	79.168			73.179	75.837	3.51	162	86.262			69.840		
58	86.389			72.286			163	66.042			64.367		
59	66.140			65.444			164	73.207	73.038	−0.23	59.661		
60	87.500			66.423	67.218	1.18	165	66.114			62.462		
61	78.246			68.885			166	60.991			54.165		
62	65.854			60.866			167	90.001	89.597	−0.45	65.217		
63	81.240			70.529			168	77.170			57.249		
64	64.290			57.249			169	95.088			67.929	69.672	2.50
65	60.271			62.106	64.739	4.07	170	73.365	72.234	−1.57	58.643		
66	92.187			64.582	70.824 ^a^	8.81	171	70.282			49.261		
67	83.173			68.420			172	69.300			61.413		
68	90.326			68.134			173	70.549			53.322	55.317	3.61
69	74.005			61.429			174	70.357	67.737	−3.87	55.179		
70	86.766			69.266	68.144	−1.65	175	82.557			56.668		
71	68.635			61.179	58.979	−3.73	176	71.336	72.986	2.26	63.146		
72	84.899			72.836	77.695	6.25	177	66.333	67.823 ^a^	2.20	52.939	50.357	−5.13
73	73.877			64.293			178	67.363			57.564		
74	85.224	85.467	0.28	65.771			179	66.274			48.973		
75	64.592			57.676			180	68.687	72.489	5.24	57.952	55.123	−5.13
76	91.184			68.336			181	73.058			49.108		
77	97.273	99.318	2.06	64.961			182	65.665			51.597		
78	95.861			70.370			183	71.546	71.487	−0.08	50.906	52.807 ^a^	3.60
79	88.812			69.859			184	73.752			43.131		
80	88.972	83.873	−6.08	74.719			185	75.222			53.835		
81	95.415	99.705 ^a^	4.30	66.087	70.222	5.89	186	73.291	75.368	2.76	58.614		
82	75.659	83.729	9.64	61.660	64.109	3.82	187	71.067			52.759		
83	75.123			64.490			188	77.819			65.671		
84	60.484			58.008			189	88.147	84.203	−4.68	66.052		
85	67.346	64.546	−4.34	57.494			190	67.586			59.613	58.962	−1.10
86	67.811			60.494			191	75.088	77.172	2.70	50.567	51.685	2.16
87	68.117			62.251			192	67.662			58.945		
88	60.852			53.986			193	69.188	69.360	0.25	58.711		
89	61.130			52.143			194	73.294	76.926 ^a^	4.72	57.909		
90	73.421			60.341			195	69.996	71.502	2.11	50.303		
91	62.968			53.689			196	73.869			51.909		
92	64.058			65.180			197	76.361	74.519	−2.47	61.428		
93	62.641			55.883			198	74.917			54.322		
94	71.336			55.614			199	73.260	72.533	−1.00	53.729		
95	65.125	70.561 ^a^	7.70	58.455			200	73.676	76.580	3.79	62.805	61.690	−1.81
96	79.861			66.412			201	68.497	70.293	2.56	58.049		
97	76.133			61.494	59.222	−3.84	202	68.262	65.828	−3.70	49.883		
98	65.547			51.651	52.414	1.46	203	78.701	78.053	−0.83	49.585		
99	65.061			56.699			204	72.909	71.865 ^a^	−1.45	60.753		
100	62.776			50.011			205	70.706	73.702	4.07	53.985		
101	72.904	71.570	−1.86	61.497	65.198 ^a^	5.68	206	76.912			55.815	59.438 ^a^	6.10
102	68.287			53.276			207	79.669	76.815 ^a^	−3.72	60.496		
103	73.465			54.758			208	74.789	75.332	0.72	55.552	55.405	−0.27
104	69.305			62.315			209	79.286	79.575	0.36	59.179	59.612	0.73

^a^ Test set.

**Table 3:** The environmental risk statistics of the three PCBs and their environmentally friendly derivative phytodegradation products.

Molecular	Phytotoxicity	Change Rate (%)	Estrogen Toxicity	Change Rate (%)	Bioconcentration	Change Rate (%)	Persistence	Change Rate (%)	Migration	Change Rate (%)
PCB-52	67.203		64.519		4.63		0.989		8.538	
52-1	90.708	34.98	65.576	1.64	3.879	−16.22	1.011	2.22	9.061	6.13
52-2	75.438	12.25	64.884	0.57	5.034	8.73	1.095	10.72	10.111	18.42
52-3	58.492	−12.96	55.954	−13.28	5.372	16.03	1.102	11.43	9.431	10.46
52-4	39.965	−40.53	69.928	8.38	1.159	−74.97	−0.831	−184.02	3.449	−59.60
P1	65.259		69.296		5.477		0.811		9.686	
P1-1	65.134	−0.19	54.331	−21.60	5.258	−4.00	0.665	−18.00	8.575	−11.47
P1-2	70.868	8.59	73.992	6.78	4.416	−19.37	0.881	8.63	8.494	−12.31
P1-3	79.052	21.14	68.901	−0.57	4.214	−23.06	0.78	−3.82	8.634	−10.86
P1-4	76.850	17.76	56.281	−18.78	4.596	−16.09	0.893	10.11	8.852	−8.61
PCB-189	88.147		66.052		5.440		1.567		11.517	
189-1	84.575	−4.05	62.105	−5.98	5.446	0.11	1.274	−18.70	10.565	−8.27
189-2	79.545	−9.76	58.150	−11.96	5.649	3.84	1.599	2.04	10.912	−5.25
189-3	84.176	−4.50	68.607	3.87	5.939	9.17	1.299	−17.10	10.702	−7.08
189-4	31.989	−63.71	59.291	−10.24	2.873	−47.19	−0.558	−135.61	4.428	−61.55
P2	71.373		49.398		3.446		1.311		4.642	
P2-1	75.601	5.92	69.555	40.81	4.439	28.82	0.694	−47.06	7.406	59.54
P2-2	67.157	−5.91	52.344	5.96	5.459	58.42	1.038	−20.82	9.928	113.87
P2-3	80.525	12.82	50.002	1.22	4.725	37.12	1.49	13.65	9.046	94.87
P2-4	86.936	21.81	58.514	18.45	5.536	60.65	1.212	−7.55	10.088	117.32
PCB-209	79.408		54.982		6.136		2.191		11.805	
209-1	85.844	8.10	50.643	−7.89	4.726	−22.98	1.85	−15.56	10.003	−15.26
209-2	77.534	−2.36	51.355	−6.60	6.204	1.11	2.079	−5.11	9.816	−16.85
209-3	61.850	−22.11	47.372	−13.84	6.079	−0.93	1.606	−26.70	10.169	−13.86
209-4	50.929	−35.86	71.124	29.36	3.190	−48.01	−0.487	−122.23	7.964	−32.54
P3	75.056		57.487		5.500		1.863		11.102	
P3-1	63.598	−15.27	48.786	−15.14	5.815	5.73	1.700	−8.75	9.383	−15.48
P3-2	79.836	6.37	58.633	1.99	5.703	3.69	1.478	−20.67	9.948	−10.39
P3-3	61.300	−18.33	58.633	1.99	5.367	−2.42	1.402	−24.75	8.608	−22.46
P3-4	79.556	6.00	52.530	−8.62	5.065	−7.91	0.949	−49.06	10.965	−1.23

**Table 4:** The total score and its estimated value of PCBs and their metabolites containing different chemical structures.

NO.		Phytotoxicity			Estrogen Toxicity		
		Total Cost	Estimated	Relative Error (%)	Total Cost	Estimated	Relative Error (%)
1	4′-OH-CB97	75.468	71.34	−5.47	66.793	55.05	−17.58
2	4′-OH-CB101	74.627	62.36	−16.44	65.252	59.60	−8.66
3	4-OH-CB107	78.598	84.60	7.64	67.165	67.39	0.34
4	4-OH-CB109	75.046	67.77	−9.70	65.372	59.92	−8.34
5	3-OH-CB118	86.550	83.53	−3.49	62.666	63.70	1.65
6	4-OH-CB148	68.485	66.70	−2.61	71.272	55.92	−21.54
7	3-OH-CB153	72.700	78.53	8.02	66.310	59.69	−9.98
8	4-OH-CB162	97.896	85.20	−12.97	61.408	64.24	4.61
9	4′-OH-CB172	80.308	66.42	−17.29	65.140	55.88	−14.22
10	4-OH-CB187	75.115	72.69	−3.23	63.185	52.85	−16.36
11	4′-OH-CB199	79.422	85.35	7.46	60.988	62.00	1.66
12	4-OH-CB202	77.024	69.99	−9.13	55.911	56.65	1.32
13	3′-MeSO_2_-CB49	72.948	67.66	−7.25	67.760	56.88	−16.06
14	4-MeSO_2_-CB64	71.831	62.72	−12.68	61.242	52.25	−14.68
15	3-MeSO_2_-CB70	86.872	78.98	−9.08	72.756	66.08	−9.18
16	3-MeSO_2_-CB110	80.192	67.57	−15.74	78.504	62.03	−20.98
17	4-MeSO_2_-CB149	83.809	81.80	−2.40	58.452	54.16	−7.34
18	4-MeSO_2_-CB174	80.324	72.89	−9.26	54.488	55.18	1.27
19	P1	66.228	65.26	−1.46	70.507	69.30	−1.72
20	P1-1	104.434	65.13	−37.63	58.480	54.33	−7.10
21	P1-2	78.201	70.87	−9.38	73.265	73.99	0.99
22	P1-3	85.788	79.05	−7.85	67.833	68.90	1.57
23	P1-4	78.552	76.85	−2.17	52.126	56.28	7.97
24	P2	77.873	71.37	−8.35	60.337	49.40	−18.13
25	P2-1	83.167	75.60	−9.10	77.099	69.56	−9.78
26	P2-2	73.239	67.16	−8.30	72.490	52.34	−27.79
27	P2-3	88.294	80.53	−8.80	65.488	50.00	−23.65
28	P2-4	80.868	86.94	7.50	64.082	58.51	−8.69
29	P3	77.512	75.06	−3.17	63.670	57.49	−9.71
30	P3-1	78.478	63.60	−18.96	53.482	48.79	−8.78
31	P3-2	76.342	79.84	4.58	63.270	58.63	−7.33
32	P3-3	76.683	61.30	−20.06	72.835	58.63	−19.50
33	P3-4	77.896	79.56	2.13	55.943	52.53	−6.10

## Supplementary Materials

The following are available online at https://www.mdpi.com/article/10.3390/toxics9090213/s1: Table S1: Estimated statistics of the environmental risk characteristics of PCBs and their transformation products.
