# Supplementary material for: Potential Environmental Risk Characteristics of PCB Transformation Products in the Environmental Medium
Source: Toxics. 2021 Sep 7;9(9):213. doi: 10.3390/toxics9090213 (PMC8472189; doi:10.3390/toxics9090213)
Supplement: Supplementary file 1 [file toxics-09-00213-s001.zip › toxics-1340093-supplementary.pdf]

toxics

MDPI

Article

Supplementary Materials: Potential Environmental Risk Characteristics of PCB Transformation Products in the Environmental Medium

Minghao Li [1,2,†], Wei He [1,†], Hao Yang [1], Shimei Sun [2,*] and Yu Li [1,*]

[1] The Moe Key Laboratory of Resources and Environmental Systems Optimization, North China Electric Power University, Beijing 102206, China; lmh66@ncepu.edu.cn (M.L.); 120202232011@ncepu.edu.cn (W.H.); 120212232080@ncepu.edu.cn (H.Y.)
[2] School of Emergency Science and Engineering, Jilin Jianzhu University, Changchun 130119, China
* Correspondence: sunshimei@jlju.edu.cn (S.S.); 50201549@ncepu.edu.cn (Y.L.)
† These authors have contributed equally to the study and they receive equal credit.

Table S1. Estimated statistics of the environmental risk characteristics of PCBs and their transformation products.

Degradation Pathway	Category	Molecular	Phytotoxicity	Change Rate (%)	Estrogen Toxicity	Change Rate (%)	Bioconcentration	Change Rate (%)	Persistence	Change Rate (%)	Migration	Change Rate (%)
Phytodegradation	Molecule	PCB-3	83.95		67.89		2.83		0.18		7.01	
	Degradation products	4 - CBA	66.11	-21.26	60.42	-11.01	4.57	61.26	0.91	421.71	9.27	32.27
	Molecule	PCB-4	73.80		63.98		3.42		0.46		7.41	
	Degradation products	2-CBA	72.80	-1.36	69.30	8.32	4.55	33.06	1.31	183.33	8.49	14.68
	Molecule	PCB-5	79.09		71.67		3.86		0.37		7.44	
	Degradation products	2,3-CBA	77.57	-1.93	69.84	-2.56	5.02	29.99	1.36	268.38	8.69	16.91
	Molecule	PCB-11	83.12		73.81		3.69		0.47		8.19	
	Degradation products	3 - CBA	64.07	-22.92	69.97	-5.20	4.69	27.28	0.73	56.87	8.29	1.22
	Molecule	PCB-31	83.42		70.01		4.16		0.70		7.98	
	Degradation products	2,5-CBA	75.28	-9.76	70.33	0.46	5.08	22.08	1.49	113.34	9.44	18.34
Microbial aerobic degradation	Molecule	PCB-97	76.13		61.49		5.19		1.09		9.40	
	Degradation products	4'-OH-CB97	71.34	-6.30	55.05	-10.48	5.35	3.10	1.13	3.85	8.90	-5.35
	Molecule	PCB-101	72.90		61.50		5.26		1.14		9.31	
	Degradation products	4'-OH-CB101	62.36	-14.46	59.60	-3.08	5.70	8.21	1.11	-2.46	10.24	10.05
	Molecule	PCB-107	84.56		70.57		5.21		1.07		10.16	
	Degradation products	4-OH-CB107	84.60	0.05	67.39	-4.50	5.37	3.05	1.09	2.06	10.34	1.72
	Molecule	PCB-109	67.58		57.75		5.32		1.01		9.65	
	Degradation products	4-OH-CB109	67.77	0.28	59.92	3.76	5.83	9.50	0.95	-6.23	8.01	-17.01
	Molecule	PCB-118	88.52		65.01		5.17		0.96		10.07	
	Degradation products	3-OH-CB118	83.53	-5.64	63.70	-2.01	5.32	2.80	0.84	-13.10	10.11	0.42
	Molecule	PCB-148	70.80		52.26		5.33		1.31		9.61	
	Degradation products	4-OH-CB148	66.70	-5.79	55.92	6.99	5.47	2.53	1.39	6.09	11.39	18.51
	Molecule	PCB-153	66.59		57.17		5.55		1.24		10.03	

	Degradation products	3-OH-CB153	78.53	17.93	59.69	4.40	5.87	5.67	1.39	12.31	9.61	-4.16
	Molecule	PCB-162	86.26		69.84		5.65		1.38		10.65	
	Degradation products	4-OH-CB162	85.20	-1.23	64.24	-8.02	6.00	6.20	1.51	9.67	11.46	7.55
	Molecule	PCB-172	69.30		61.41		6.05		1.58		11.06	
	Degradation products	4'-OH-CB172	66.42	-4.16	55.88	-9.01	5.84	-3.54	1.69	7.04	12.04	8.86
	Molecule	PCB-187	71.07		52.76		5.70		1.56		12.00	
	Degradation products	4-OH-CB187	72.69	2.28	52.85	0.18	5.92	3.72	1.60	2.11	10.80	-10.01
	Molecule	PCB-199	73.26		53.73		5.93		1.74		11.10	
	Degradation products	4'-OH-CB199	85.35	16.50	62.00	15.40	5.61	-5.40	1.08	-37.71	8.92	-19.68
	Molecule	PCB-202	68.26		49.88		5.81		1.81		10.30	
	Degradation products	4-OH-CB202	69.99	2.53	56.65	13.56	5.45	-6.13	2.08	14.67	10.24	-0.66
Microbial anaerobic degradation	Molecule	PCB-90	73.42		60.34		5.12		1.14		9.63	
	Degradation products	PCB-49	71.32	-2.86	61.96	2.68	4.58	-10.56	0.97	-14.96	8.57	-11.03
	Degradation products	PCB-68	90.33	23.02	68.13	12.91	4.73	-7.55	0.86	-24.30	8.74	-9.24
	Molecule	PCB-91	62.97		53.69		5.05		1.05		9.33	
	Degradation products	PCB-51	63.43	0.74	52.78	-1.70	4.55	-9.93	0.90	-14.24	8.44	-9.58
	Molecule	PCB-92	64.06		65.18		5.25		1.16		9.31	
	Degradation products	PCB-52	67.20	4.91	64.52	-1.01	4.71	-10.30	0.99	-14.74	8.54	-8.28
	Degradation products	PCB-72	84.90	32.53	72.84	11.75	4.73	-9.99	0.99	-14.91	8.64	-7.23
	Molecule	PCB-95	65.13		58.46		5.13		1.05		8.99	
	Degradation products	PCB-53	68.36	4.96	66.01	12.92	4.56	-11.08	0.92	-11.75	8.24	-8.32
	Molecule	PCB-99	65.06		56.70		5.13		1.12		9.63	
	Degradation products	PCB-47	65.82	1.16	57.20	0.88	4.63	-9.64	0.83	-25.45	8.89	-7.66
	Molecule	PCB-101	72.90		61.50		5.26		1.14		9.31	
	Degradation products	PCB-49	71.32	-2.17	61.96	0.75	4.58	-13.09	0.97	-15.19	8.57	-7.90
	Molecule	PCB-102	68.29		53.28		5.12		1.11		10.43	
	Degradation products	PCB-51	63.43	-7.11	52.78	-0.93	4.55	-11.09	0.90	-19.04	8.44	-19.10
	Molecule	PCB-130	74.55		62.57		5.48		1.29		10.33	
	Degradation products	PCB-90	73.42	-1.51	60.34	-3.57	5.12	-6.58	1.14	-12.01	9.63	-6.77
	Molecule	PCB-132	66.79		54.86		5.32		1.31		11.04	
	Degradation products	PCB-91	62.97	-5.72	53.69	-2.13	5.05	-4.96	1.05	-19.97	9.33	-15.48
	Molecule	PCB-135	64.86		59.01		5.35		1.33		9.90	
	Degradation products	PCB-94	71.34	9.98	55.61	-5.76	5.08	-5.03	1.09	-17.74	8.94	-9.69
	Molecule	PCB-137	67.12		58.50		5.62		1.29		10.37	
	Degradation products	PCB-90	73.42	9.40	60.34	3.15	5.12	-8.95	1.14	-11.66	9.63	-7.13
	Degradation products	PCB-99	65.06	-3.06	56.70	-3.08	5.13	-8.78	1.12	-13.22	9.63	-7.19
	Molecule	PCB-138	68.89		57.87		5.48		1.24		10.07	

	Degradation products	PCB-99	65.06	-5.56	56.70	-2.02	5.13	-6.43	1.12	-10.22	9.63	-4.42
	Molecule	PCB-146	75.03		60.76		5.55		1.38		10.03	
	Degradation products	PCB-90	73.42	-2.15	60.34	-0.70	5.12	-7.84	1.14	-17.50	9.63	-3.99
	Molecule	PCB-147	65.47		52.28		5.27		1.28		9.95	
	Degradation products	PCB-91	62.97	-3.82	53.69	2.69	5.05	-4.08	1.05	-18.41	9.33	-6.15
	Molecule	PCB-149	68.51		54.16		5.34		1.33		9.71	
	Degradation products	PCB-102	68.29	-0.33	53.28	-1.62	5.12	-4.05	1.11	-16.75	10.43	7.47
	Molecule	PCB-151	67.63		57.03		5.49		1.25		9.65	
	Degradation products	PCB-95	65.13	-3.70	58.46	2.50	5.13	-6.65	1.05	-16.17	8.99	-6.83
	Molecule	PCB-153	66.59		57.17		5.55		1.24		10.03	
	Degradation products	PCB-99	65.06	-2.29	56.70	-0.83	5.13	-7.67	1.12	-9.64	9.63	-3.99
	Molecule	PCB-154	67.69		50.53		5.36		1.23		9.59	
	Degradation products	PCB-100	62.78	-7.27	50.01	-1.03	5.02	-6.36	1.01	-18.00	8.91	-7.09
	Molecule	PCB-170	73.37		58.64		5.96		1.55		11.47	
	Degradation products	PCB-130	74.55	1.61	62.57	6.70	5.48	-8.17	1.29	-16.76	10.33	-9.91
	Degradation products	PCB-137	67.12	-8.52	58.50	-0.24	5.62	-5.77	1.29	-17.09	10.37	-9.56
	Degradation products	PCB-138	68.89	-6.10	57.87	-1.33	5.48	-8.13	1.24	-19.86	10.07	-12.19
	Molecule	PCB-174	70.36		55.18		5.57		1.54		10.60	
	Degradation products	PCB-149	68.51	-2.63	54.16	-1.85	5.34	-4.24	1.33	-13.80	9.71	-8.38
	Molecule	PCB-180	68.69		57.95		6.04		1.53		10.77	
	Degradation products	PCB-153	66.59	-3.06	57.17	-1.34	5.55	-8.10	1.24	-19.12	10.03	-6.92
	Degradation products	PCB-146	75.03	9.24	60.76	4.85	5.55	-8.11	1.38	-9.82	10.03	-6.87
	Molecule	PCB-183	71.55		50.91		5.75		1.55		10.35	
	Degradation products	PCB-154	67.69	-5.38	50.53	-0.73	5.36	-6.71	1.23	-20.72	9.59	-7.36
	Molecule	PCB-187	71.07		52.76		5.70		1.56		12.00	
	Degradation products	PCB-149	68.51	-3.60	54.16	2.65	5.34	-6.45	1.33	-14.90	9.71	-19.09
	Molecule	PCB-49	71.32		61.96		4.58		0.97		8.57	
	Degradation products	3'-MeSO2-CB49	67.66	-5.14	56.88	-8.19	5.46	19.39	0.91	-5.69	10.24	19.44
	Molecule	PCB-64	64.29		57.25		4.78		0.81		8.69	
	Degradation products	4-MeSO2-CB64	62.72	-2.44	52.25	-8.74	5.27	10.23	1.15	42.10	9.61	10.50
Biometabolism	Molecule	PCB-70	86.77		69.27		4.77		0.96		9.13	
	Degradation products	3-MeSO2-CB70	78.98	-8.97	66.08	-4.61	5.57	16.77	1.18	22.72	10.99	20.42
	Molecule	PCB-110	68.63		61.74		5.12		1.08		9.84	
	Degradation products	3-MeSO2-CB110	67.57	-1.56	62.03	0.48	4.98	-2.75	1.17	8.45	9.56	-2.92

	Molecule	PCB-149	68.51		53.15		5.34		1.33		9.71	
	Degradation products	4-MeSO2-CB149	81.80	19.40	54.16	1.89	5.34	0.13	1.63	22.09	10.56	8.82
	Molecule	PCB-174	70.36		56.80		5.57		1.54		10.60	
	Degradation products	4-MeSO2-CB174	72.89	3.60	55.18	-2.86	5.32	-4.45	1.72	11.66	11.12	4.95
	Molecule	PCB-47	65.82		57.20		4.63		0.83		8.89	
	Degradation products	PCB-15	89.55	36.06	65.04	13.72	3.59	-22.50	0.33	-60.70	7.87	-11.43
	Molecule	PCB-40	73.33		65.30		4.56		0.90		9.17	
Photodegradation	Degradation products	PCB-11	83.12	13.35	73.81	13.03	3.69	-19.16	0.47	-48.11	8.19	-10.68
	Molecule	PCB-101	72.90		61.50		5.26		1.14		9.31	
	Degradation products	PCB-70	86.77	19.01	69.27	12.63	4.64	-11.85	0.96	-15.36	9.13	-1.89
	Molecule	PCB-171	70.28		49.26		5.73		1.53		10.72	
	Degradation products	PCB-35	86.14	22.56	69.104	0.28	4.41	-22.93	0.62	-59.57	8.99	-16.20
